# Factors contributing to psychological distress in the working population, with a special reference to gender difference

**DOI:** 10.1186/s12889-021-10560-y

**Published:** 2021-03-29

**Authors:** Satu Viertiö, Olli Kiviruusu, Maarit Piirtola, Jaakko Kaprio, Tellervo Korhonen, Mauri Marttunen, Jaana Suvisaari

**Affiliations:** 1 Department of Public Health and Welfare, Finnish Institute for Health and Welfare in Finland (THL), Helsinki, Finland; 2 Adolescent Psychiatry, University of Helsinki and Helsinki University Hospital, Helsinki, Finland; 3 University of Helsinki, Institute for Molecular Medicine Finland (FIMM), Helsinki, Finland; 4 Department of Public Health, University of Helsinki, Helsinki, Finland

**Keywords:** Psychological distress, Work-family conflict, Gender, Nationally representative study

## Abstract

**Background:**

Psychological distress refers to non-specific symptoms of stress, anxiety and depression, and it is more common in women. Our aim was to investigate factors contributing to psychological distress in the working population, with a special reference to gender differences.

**Methods:**

We used questionnaire data from the nationally representative Finnish Regional Health and Well-being Study (ATH) collected in the years 2012–2016 (target population participants aged 20 +, *n* = 96,668, response rate 53%), restricting the current analysis to those persons who were working full-time and under 65 of age (*n* = 34,468). Psychological distress was assessed using the Mental Health Inventory-5 (MHI-5) (cut-off value <=52). We studied the following factors potentially associated with psychological distress: sociodemographic factors, living alone, having children under18 years of age, lifestyle-related factors, social support, helping others outside of the home and work-related factors. We used logistic regression analysis to examine association between having work-family conflict with the likelihood for psychological distress. We first performed the models separately for men and women. Then interaction by gender was tested in the combined data for those independent variables where gender differences appeared probable in the analyses conducted separately for men and women.

**Results:**

Women reported more psychological distress than men (11.0% vs. 8.8%, respectively, *p* < 0.0001). Loneliness, job dissatisfaction and family-work conflict were associated with the largest risk of psychological distress. Having children, active participation, being able to successfully combine work and family roles, and social support were found to be protective factors. A significant interaction with gender was found in only two variables: ignoring family due to being absorbed in one’s work was associated with distress in women (OR 1.30 (95% CI 1.00–1.70), and mental strain of work in men (OR 2.71 (95% CI 1.66–4.41).

**Conclusions:**

Satisfying work, family life and being able to successfully combine the two are important sources of psychological well-being for both genders in the working population.

**Supplementary Information:**

The online version contains supplementary material available at 10.1186/s12889-021-10560-y.

## Background

Psychological distress refers to non-specific symptoms of stress, anxiety and depression. High levels of psychological distress are indicative of impaired mental health and may reflect common mental disorders, like depressive and anxiety disorders [1]. It is commonly measured with self-report rating scales like the General Health Questionnaire [2] or MHI-5, derived from the RAND-36 questionnaire [3]. As psychological distress also predicts sickness absences and work disability among the working-age population [4, 5], it is important to understand the factors that contribute to psychological distress among those who are working.

According to previous studies, women in the Western world are more prone to psychological distress, depression and anxiety than men [6–9]. Proposed explanations for the gender difference include biological, psychological and social risk factors [10, 11]. Social factors involve, e.g. different societal roles and expectations for men and women. The roles at work and in the family as well as the challenges in combining them may be one factor contributing to gender differences [12–14]. However, the combination may also create more content and satisfaction in life, including with respect to possible gender differences [15].

### Work-to-family conflict, family-to-work conflict and work-family enrichment load

Contradictions between work and family, a work-family conflict involves two separate, but related domains. One is work-to-family conflict, also called work-family interference or work interference with family, which occurs when participation in family life is made more difficult by work-related demands [16]. Family-to-work conflict, also called family-work interference or family interference with work, occurs when family life interferes with work [17]. In contrast, work-to-family enrichment means that the experiences at work improve one’s performance and satisfaction within the family [15, 18]. Role accumulation theory claims that multiple roles and meaningful content in life create a positive conception of oneself [19].

Work-family conflict has been found to be more common in women, although the gender difference in European countries is currently small [20]. Women still perform most of the domestic work in families [21]. Both having children and providing informal care to elderly relatives may increase the experience of work-family conflict [20]. One negative consequence of work-family conflict suggested by previous research is that women may reduce their contribution in work domain and that in turn may hinder career advancement [13]. According to European statistics, when the time spent travelling between home and the workplace and doing unpaid work are taken into account, women work on average 64 h a week compared to 53 h for men. Women spend on average 26 h taking care of children and elderly relatives, whereas men spend only 9 h [22]. It seems that especially during parenting, women have more problems in coordinating work and family life [17, 23, 24].

According to a 2010 European Social Survey, mothers and higher educated employees report the highest rates of work-family conflict [25]. Highly educated parents tend to experience more work-family conflict than less educated parents because of longer working days and greater difficulty in separating work from leisure time. A work position where an individual has much authority and responsibility to make decisions, has been found to increase the risk of psychological distress [26].

### Other work-related factors

According to a meta-analysis [27], a low level of job satisfaction is associated with a higher risk of psychological distress, burnout, anxiety and depression. Significant gender differences in job satisfaction have not been found, although women are less likely to work in managerial jobs and their salary is commonly lower [28–30].

Mental and physical work strain may affect mental health. Mental strain is common in human service work, but while working in these professions may increase the risk of emotional exhaustion and psychological distress, it may also provide meaning in work [31]. Physical work strain has been found to have a stronger effect on mental health in men than in women [32].

### Social support, loneliness and other social environmental factors

Perceived social support refers to a person’s sense that emotional or practical support is available from others when needed. A lack of social support from one’s partner and close relatives, parents and friends is a risk factor for psychological distress [33]. There are indications that it operates in different ways for men and women [34], such as the fact that emotional support is more protective against depression for women than for men [33]. Women benefit from support more than men in both work and family contexts [35] and have more supportive networks than men do [36]. In contrast, women seem to receive less support from their spouses than men do from theirs [37].

Social support, especially emotional support, is often related to leisure-time activities, such as hobbies or cultural activities, and women tend to gain more benefit from social participation than men [33, 38]. It seems that leisure-time activities are associated with better mental health, especially when they include social contacts, and this is true particularly for men [39].

Emotional loneliness is the absence of someone to turn to in times of need, while social loneliness is the absence of a social network [40]. Loneliness, which women report experiencing more commonly than men in the general population, co-occurs with mental disorders and psychological distress [41, 42], and its association is partly independent of perceived social support [43]. Accordingly, emotional loneliness is more strongly associated with distress and mental disorders than social loneliness [42]. Among college students, loneliness has a greater impact on women’s mental health than it does on men’s [44], but differences between genders have not been found among community dwelling adults [45].

Marital status appears to be a significant feature in loneliness. Marriage, compared to widowhood and divorce, has been found to be associated with better mental well-being in both genders [46, 47], while becoming widowed has more long-term effects among men than among women [48]. Living alone has mostly been associated with a greater risk of experiencing mental health problems [49], in some studies particularly among men [50], and men especially experience greater mortality rates from mental disorders than do women [51]. However, findings, especially among elderly people, have also shown that living alone is not associated with reduced emotional well-being [52] or psychological distress [53].

Studies on parenting and mental health have mainly focused on how parental stress and depression affects children [54] and a depressed parent’s behaviour as a parent [55]. Parenthood itself as a risk or protective factor has been studied less often. Some studies have found that parenthood is associated with less mental health problems [56, 57], whereas no association between mental health and parenthood has been found when different types of family statuses, like single parenthood and divorce or living alone, have been taken into account [58].

### Other factors

Harmful lifestyle factors, like smoking and heavy alcohol intake, have been found to be associated with an increased risk for depressive symptoms [59–62]. Cigarette smoking is more common in lower socioeconomic groups and among people with mental disorders or psychological distress [63–65]. Moreover, the association between smoking and psychological distress appears to have become stronger in recent decades.

Financial difficulties have been found to be a risk factor for reduced mental health [66, 67]. It is not just poverty that causes psychological distress, but also the stigma associated with receiving public assistance [68]. The risk of suffering common mental disorders, e.g. depression and anxiety disorders, among men and women appears different when viewed by income category; women’s risk is greater than men’s risk in all other categories except the lowest one, [69], whereas financial difficulties in covering household costs seem to have equal negative effects on mental health both in men and women [70, 71].

Informal caregiving, e.g. helping elderly parents, may increase psychological distress, and a recent study has found this to be true for women but not for men [72]. However, other studies have not found an association between informal caregiving and mental health [73].

### Aims of the study

There is a growing concern in Finland related to increasing rates and widening gender gap in sickness absences and disability pensions related to mental disorders [74, 75] Therefore, it is important to study factors contributing to psychological distress in the working population and to identify factors that may relate to these gender differences.

The aim of the current study was to investigate factors contributing to psychological distress among those working full-time, with a special reference to gender differences. A large and representative general population-based survey sample was used, and variables were chosen in the regression models so that they would cover the most important domains that potentially influence psychological distress. Our hypothesis was that factors related to work, family and conflicts in their coordination would be particularly relevant for gender differences associated with psychological distress in the general population.

## Methods

### Design and population

**Fig. 1:**
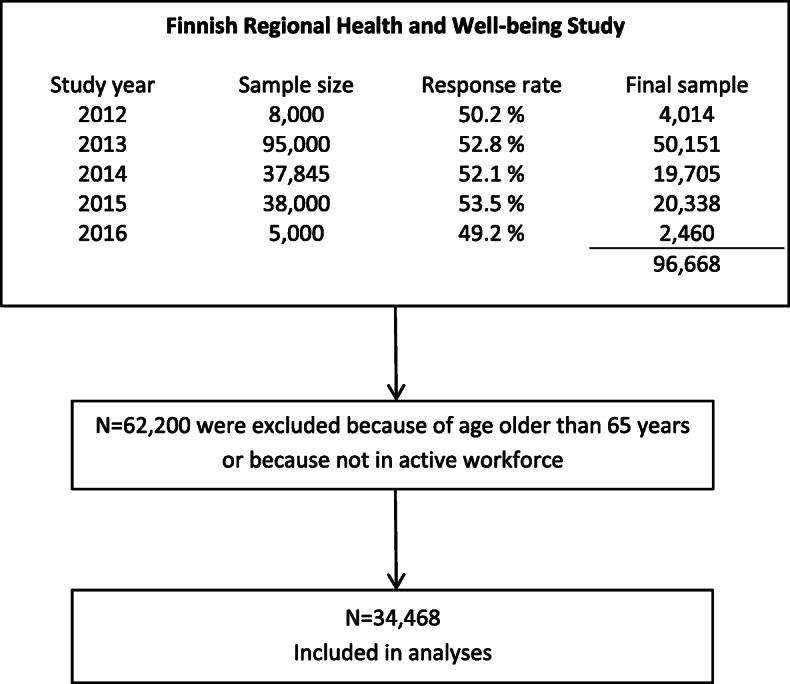
Flow chart of the sample sizes and response rates to the Finnish Regional Health and Well-being Study and participants in the present study

The Regional Health, Wellbeing and Service Use Study (ATH) was set out to provide regional (regions or municipalities) information for monitoring on factors affecting health, wellbeing and service use in Finland. Several questions derived from the study are used as national indicators and reported in the Sotkanet portal (sotkanet.fi). Sotkanet portal provides demographic indicators across Finland and Europe on health, welfare and functioning of the service-system. The survey was targeted at the population of Finland aged 20 years or over, implemented annually from 2010 to 2016. Since 2017, the survey has been called Finsote and its content has changed slightly. A stratified random sampling design, described in detail by Härkänen et al. [76], was used, and the sampling was done without replacement. The sample was drawn from the Finnish Population Register. Participants were informed about the purposes of the survey, as well as about data security. In the selection phase of the new sample, there is the exclusion of persons who have been included into the samples of the ATH survey in previous years. Inverse probability weighting was used to account for missing data [76]. Data used in the present study are from nationally representative samples collected in the years 2012–2016 (*n* = 96,668). In the present analyses, we used answers from participants aged 65 years or younger and who were working full-time (*n* = 34,468) (Fig. 1). The respondents returned the questionnaire either by mail or online, and it was possible to answer in four languages: Finnish, Swedish, Russian and English. ATH study was approved by the Coordinating Ethics Committee of Finnish Institute for Health and Welfare (THL) in 2010 (approval number THL107/6.01.00/2010).

### Methods

The questionnaire was designed by a group of scientists and specialists at the Finnish Institute for Health and Welfare with members from Finnish Institute of Occupational Health, the Social Insurance Institution of Finland and Institute of Criminology and Legal Policy. The writers of the article chose the questions into the current study from the original ATH study questionnaire and they are found as an additional file.

Psychological distress was assessed using the MHI-5 [3, 5]. MHI-5 is derived from the RAND-36 questionnaire [3], which is a widely used self-report instrument to measure health-related quality of life. It includes eight concepts: physical functioning, bodily pain, role limitations due to physical health problems, role limitations due to personal or emotional problems, emotional well-being, social functioning, energy/fatigue, and general health perceptions [3]. The MHI-5 consists of five questions: ‘How much of the time during the last month have you: 1) been a very nervous person, 2) felt downhearted and blue, 3) felt calm and peaceful, 4) felt so down in the dumps that nothing could cheer you up and 5) been a happy person? The six possible responses to the questions were scored between 1 and 6. Items 3 and 5 ask about positive feelings and their scoring was done in reverse. All scores were then converted to fit a range from 0 to 100, with low scores indicating more psychological distress.

There is not one established cut-off point for measuring clinically significant psychological distress by MHI-5 [1]. We used the cut-off of 52 points, derived from the Eurobarometer survey in 2002 [77], in which Finland participated as well. The same cut-off score has been used throughout the history of the ATH study. ATH started regionally already in 2009. Cronbach’s alpha, which is a measure of internal consistency, was 0.85.

The variables chosen as potential risk or protective factors for psychological distress are presented in Table 1; the table format is adapted from Abbas et al. [78].

**Table 1:** Protective and risk factors for psychological distress in the research data according to previous literature

**Protective factors**	**Conceptualisation in the survey questionnaire**	**Reference**
Sociodemographic	Being married Having children under 18 years of age	Ben-Zur 2018 [46] Scott et al. 2010 [47] Helbig et al. 2006 [56] McKenzie et al. 2012 [57]
Social support	Being active in societies, hobby groups, etc. Having other people to give practical help Having other people to give emotional support	Kendler et al. 2005 [33] Amagasa et al. 2017 [38] Takeda et al. 2015 [39] Perrewe et al. 2002 [35] Belle 1988 [36]
Work-family enrichment	Being able to forget the work at home Having more energy to be with children when also going to work	Greenhaus et al. 2006 [15] Xu et al. 2018 [18]
**Risk factors**	**Conceptualisation in the survey questionnaire**	
Sociodemographic	Being divorced Being widowed Living alone	Ben-Zur 2018 [46] Scott et al. 2010 [47] Kaprio et al. 1987 [48] Pulkki-Råback et al. 2012 [49] Joutsenniemi et al. 2005 [50]
Lack of social support	Loneliness	Beutel et al. 2017 [41] Hyland et al. 2019 [42]
Financial difficulties	Having difficulties in covering household costs	Nagasu et al. 2019 [66] Holden et al. 2016 [67]
Informal caregiving	Helping parents outside the home Helping children outside the home Helping other people outside the home	Lacey et al. 2018 [72]
Job related factors	Low job satisfaction High physical strain of work	Faragher et al. 2003 [27] Hiesinger et al. 2019 [32]
Work-to-family conflict	Feeling of neglecting domestic issues because of the work Ignoring family when wholly absorbed in the work Feeling inadequacy as a parent	Remery et al. 2019 [20]
Family-to-work conflict	Finding it difficult to concentrate of work because of domestic issues	Marchand et al. 2016 [13] Byron 2005 [17] Lunau et al. 2014 [23] Eby et al. 2005 [24]
Harmful lifestyle factors	Alcohol Smoking	Ranjit et al. 2019 [59] Grant et al. 2004 [60] Li et al. 2019 [61] Berg et al. 2019 [62]

### Work-related variables

Potential work-family conflict was assessed using the following question: ‘Are the following statements about home and work accurate for you?’ The respondents were then asked to agree or disagree with six statements. One statement was considered protective: ‘I have more energy to be with the children when I also go to work’. Another statement was considered neutral: ‘When I come home, I stop thinking about my work’. Work interference with family was assessed via three statements: ‘I feel I am neglecting domestic issues because of my work’, ‘I sometimes ignore my family when I am wholly absorbed in my work’ and ‘I feel inadequacy as a parent’. Family interference with work was assessed with the statement ‘I often find it difficult to concentrate on my work because of domestic issues’. The answers were divided into two classes: agree and disagree/cannot say.

Job satisfaction was measured using the following question: ‘How satisfied are you with your present work?’ The responses were divided into four categories: extremely satisfied, fairly satisfied, neither satisfied nor dissatisfied, and fairly/extremely dissatisfied. We merged classes 4 and 5 due to too few observations. Mental and physical strain of one’s work was measured using the following question: ‘What is/was you most recent job like (physically and mentally)?’ Answers were divided into three categories: low strain (light, fairly light), moderate strain (a bit or quite strenuous) and high strain (very strenuous).

Questions related to job satisfaction and strain and work-family and family-work conflict are from the Finnish Quality of Work Life Surveys 1977–2008 [79].

### Sociodemographic and other non-work-related variables

The age of the participants was divided into three categories: 20–34 years, 35–49 years and 50–65 years. Education was likewise divided into three categories: less or equal to 12 years, 13–16 years and 17 years or more. Marital status was categorised as married/cohabiting, separated/divorced/widowed and single. Having children under 18 years of age was divided into a yes or no category. Living alone was likewise categorised based on a response of yes or no. All the participants in the analyses had a full-time job.

Those who reported that they smoke on a daily basis were classified as smokers, others as non-smokers. Alcohol consumption was assessed using the Alcohol Use Disorders Identification Test (AUDIT-C) [80], which included the following questions: ‘How often do you have a drink containing alcohol?’, ‘How many standard drinks of alcohol do you have on a typical day when you are drinking?’, ‘How often do you have 5 or more drinks on one occasion?’. A total score of six or more for men and five or more for women indicated at-risk drinking.

Subjectively experienced loneliness was divided into two categories: never/seldom/sometimes and often/all the time. Participation in leisure-time activities, such as hobby groups, societies and so forth, was categorised as regular or never/sometimes. Helping parents, children or other people outside the home regularly was defined as occurring at least once or twice a month.

Whether or not respondents received practical support when needed was identified using the following question: ‘Who will provide practical help when you need it?’ For emotional support, the question was worded as follows: ‘Who do you believe truly cares about you, whatever may happen?’. Possible helpers or carers were partner, other next of kin, close friend, close colleague, close neighbours and other persons in close proximity to you. The answers were divided into three categories: no one helps or cares, 1–2 persons help or care, and 3–6 persons help or care. Financial problems were assessed with the question ‘How difficult or easy is it to cover your living costs?’. The responses were divided into two categories: very difficult/fairly difficult and fairly easy/very easy.

### Statistical analysis

Analyses were conducted using the SAS Enterprise Guide 7.1 [81] and SUDAAN Release 11.0.3 [82]. Weights were used to take into account the sampling design and non-participation, so that the results would be representative of the Finnish working-age population. We calculated the distribution of sociodemographic variables, other non-work-related variables and work-related variables for both genders. Gender differences in the categorical variables were tested using the two-tailed χ2 test. Next we calculated the prevalence of psychological distress in both genders according to the levels of the study variables.

We used logistic regression analysis to examine association between having family-to-work or work-to-family conflict with the likelihood for psychological distress (cut-off value of MHI-5 < =52). Dependent variable in the model was psychological distress (yes/ no) measured with MHI-5 and independent variables, i.e. sociodemographic factors, other non-work-related factors and work-related factors were included in the model simultaneously. We first performed the models separately for men and women. Then interaction by gender was tested in the combined data for those independent variables where gender differences appeared probable in the analyses conducted separately for men and women.

## Results

### Characteristics of study variables in men and women

Characteristics of the study variables are presented in Table 2 for men and women separately. Women reported more distress than men did, and with most of the other variables also exhibited a statistically significant gender difference. We found no gender difference for the variables living alone, having school-age children or being active in societies and hobby groups. Men and women differed in all but one work-family conflict: ‘I have more energy to be with the children when I also go to work’.

**Table 2:** Characteristics of the sample by gender (numbers, percentages, means, and 95% confidence intervals)

	Men (*n* = 15,140)	Women (*n* = 18,112)	*P* value
	n (%)	n (%)	
MHI-5 (cut-off <=52 points)	1321 (8.8)	1984 (11.0)	< 0.0001
MHI-5 mean (95% CI)	75.8 (75.6–76.1)	74.4 (74.1–74.6)	
Age (years)			
20–34	3496 (28.7)	3852 (24.5)	< 0.0001
35–49	5834 (39.7)	6791 (38.2)	
50–65	6322 (31.6)	8087 (37.4)	
Age (mean)	42.4 (42.2–42.6)	43.9 (43.7–44.0)	
Education (years)			
< = 12	6224 (43.0)	4793 (26.9)	< 0.0001
13–16	5367 (33.6)	8089 (43.4)	
17+	4061 (23.4)	5849 (29.7)	
Marital status			
Married/cohabiting	12,016 (76.1)	13,495 (72.9)	< 0.0001
Separated/divorced/widowed	1017 (6.7)	2255 (12.3)	
Single	2105 (17.2)	2360 (14.8)	
Lives alone			
Yes	2011 (14.5)	2703 (14.9)	0.263
No	13,641 (85.5)	16,028 (85.1)	
Has children under 18 years of age			
< 3 years old	1605 (21.9)	840 (9.7)	< 0.0001
3 to 6 years old	2032 (26.2)	1903 (20.2)	< 0.0001
7 to 17 years old	4459 (44.8)	5277 (44.2)	0.373
At-risk drinking (AUDIT-C)^a^	5678 (37.5)	4087 (22.8)	< 0.0001
Current smoker	2350 (25.6)	2346 (23.0)	< 0.0001
Loneliness			
Never/seldom/sometimes	14,424 (94.1)	16,988 (92.7)	< 0.0001
Quite often/all the time	810 (5.9)	1273 (7.3)	
Active member in societies, hobby groups, etc.	4609 (28.9)	5318 (28.1)	0.0962
Difficulty in covering household costs	3583 (25.2)	4567 (26.5)	0.011
How many persons give you practical help?			
No one	217 (1.6)	201 (1.2)	6.64
1–2 persons	8303 (54.4)	9697 (53.5)	0.0013
3–6 persons	6412 (43.0)	7957 (45.3)	
How many persons give you emotional support?			
No one	150 (1.1)	146 (0.8)	< 0.0001
1–2 persons	9725 (63.8)	9639 (53.4)	
3–6 persons	5096 (35.1)	8108 (45.8)	
Helping someone outside the household regularly, at least once a month			
Parent	4629 (41.3)	5846 (44.3)	< 0.0001
Child	3204 (28.6)	4691 (36.6)	< 0.0001
Other	3394 (32.5)	4921 (39.3)	< 0.0001
Work satisfaction			
Extremely satisfied	2889 (18.6)	3883 (20.9)	< 0.0001
Fairly satisfied	8664 (56.0)	10,420 (56.6)	
Neither satisfied nor dissatisfied	2508 (16.8)	2547 (14.2)	
Fairly - extremely dissatisfied	1233 (8.6)	1431 (8.2)	
Mental strain of work			
Low	2359 (16.7)	2202 (12.9)	< 0.0001
Moderate	10,624 (69.2)	13,118 (72.0)	
High	2089 (14.0)	2708 (15.1)	
Physical strain of work			
Low	7796 (47.8)	9710 (52.1)	< 0.0001
Moderate	6299 (44.0)	7121 (40.9)	
High	1080 (8.2)	1142 (7.0)	
When I come home, I stop thinking about my work ^b^	8147 (55.4)	10,709 (59.6)	< 0.0001
I feel I am neglecting domestic issues because of my work^b^	5502 (36.2)	7126 (39.2)	< 0.0001
I sometimes ignore my family when I am wholly absorbed in my work^b^	4290 (27.1)	4512 (24.1)	< 0.0001
I often find it difficult to concentrate on my work because of domestic issues^b^	1575 (10.9)	1718 (10.1)	0.020
I have more energy to be with the children when I also go to work^b^	5419 (34.9)	6159 (34.5)	0.428
I feel inadequacy as a parent^b^	2324 (15.1)	3538 (20.0)	< 0.0001

^a^At-risk drinking is assessed as AUDIT-C, men > = 6 points and women > = 5 points

^b^N indicates the number of yes answers

### Associations of study variables with psychological distress by gender

Cross-tabulation of different variables with psychological distress in men and women are presented in Table 3. Most of the independent variables were associated with psychological distress, but with some gender differences. Education was associated with psychological distress only in women, with less education being associated with more distress. Helping a parent or a child outside of a person’s own household was associated with less distress, but statistically significantly only in men helping a parent and in women helping a child. Helping somebody other than one’s own child or parent was associated with more distress, but statistically significantly only in women.

**Table 3:** Associations of study variables with psychological distress by gender (MHI-5 cut-off point <=52) (%)

%	Men (*N* = 15,059)			Women (*N* = 18,050)		
	Psychological distress (*N* = 1321)	No psychological distress (*N* = 13,738)	*P* value	Psychological distress (*N* = 1984)	No psychological distress (*N* = 16,066)	*P* value
	% (N)	% (N)		% (N)	% (N)	
**Age group**						
20–34 (21.4)	12.0 (388)	88.0 (2977)	< 0.0001	15.6 (554)	84.4 (3138)	< 0.0001
35–49 (36.7)	9.6 (507)	90.4 (5103)		10.9 (688)	89.1 (5865)	
50–65 (41.9)	7.5 (426)	92.5 (5658)		9.9 (742)	90.1 (7063)	
**Education years**						
< =12 (32.0)	10.0 (541)	90.0 (5355)	0.0756	13.1 (548)	86.9 (3992)	0.0013
13–16 (39.1)	9.9 (464)	90.1 (4753)		11.5 (844)	88.5 (6982)	
17+ (28.9)	8.6 (316)	91.4 (3630)		10.7 (592)	89.3 (5093)	
**Marital status**						
Married/cohabiting (76.7)	8.1 (879)	91.9 (10,743)	< 0.0001	10.0 (1238)	90.0 (11,814)	< 0.0001
Separated/divorced/widowed (9.9)	14.2 (123)	85.8 (11,622)		14.7 (308)	85.3 (1879)	
Single (13.4)	14.5 (280)	85.5 (1746)		16.7 (365)	83.3 (1895)	
**Lives alone**						
Yes (13.7)	14.7 (262)	85.3 (1690)	< 0.0001	15.4 (385)	84.6 (2243)	< 0.0001
No (86.3)	8.7 (1059)	91.3 (12,048)		11.0 (1599)	89.0 (13,824)	
**Has children under 18 years of age**						
Yes (53.1)	8.6 (498)	91.4 (5565)	< 0.0001	11.1 (674)	88.9 (5699)	0.0023
No (46.9)	11.6 (495)	88.4 (4232)		12.9 (777)	87.1 (5604)	
**At-risk drinking**^a^						
Yes (28.4)	12.0 (606)	88.0 (4930)	< 0.0001	15.2 (574)	84.8 (3414)	< 0.0001
No (71.6)	8.1 (705)	91.9 (8808)		10.6 (1410)	89.4 (12,653)	
**Current smoker**						
Yes (22.3)	13.6 (291)	86.4 (1987)	< 0.0001	19.2 (411)	80.8 (1869)	< 0.0001
No (77.7)	8.6 (646)	91.4 (6908)		10.7 (900)	89.3 (7503)	
**Loneliness**						
Never/seldom/sometimes (93.8)	7.1 (933)	92.9 (13,285)	< 0.0001	8.8 (1394)	91.2 (15,381)	< 0.0001
Often/all the time (6.2)	49.3 (385)	50.7 (409)		48.1 (587)	51.9 (669)	
**Active member in societies, hobby groups, etc.**						
Yes (29.5)	6.9 (283)	93.1 (4182)	< 0.0001	8.0 (397)	92.0 (4775)	< 0.0001
No (70.5)	10.7 (1020)	89.3 (9367)		13.1 (1553)	86.9 (11,021)	
**How difficult or easy is it to cover household costs?**						
Fairly difficult/very difficult (24.0)	29.0 (251)	71.0 (655)	< 0.0001	29.2 (369)	70.8 (914)	< 0.0001
Fairly easy /very easy (76.0)	8.2 (1063)	91.8 (12,998)		10.1 (1592)	89.9 (15,013)	
**How many persons give you practical help?**						
No one (1.3)	39.8 (77)	60.2 (128)	< 0.0001	40.8 (75)	59.2 (119)	< 0.0001
1–2 persons (54.9)	11.8 (863)	88.2 (7215)		13.9 (1223)	86.1 (8161)	
3–6 persons (43.8)	5.8 (331)	94.2 (5890)		8.1 (593)	91.9 (7187)	
**How many persons give you emotional support?**						
No one (0.9)	40.9 (54)	59.1 (85)	< 0.0001	45.2 (61)	54.8 (79)	< 0.0001
1–2 persons (58.9)	10.9 (931)	89.1 (8531)		13.8 (1206)	86.2 (8120)	
3–6 persons (40.2)	6.2 (288)	93.8 (4646)		8.3 (628)	91.7 (7291)	
**Helping a parent outside the household regularly, at least once a month**						
Yes (42.9)	9.1 (382)	90.9 (4099)	0.0077	11.7 (634)	88.3 (5067)	0.1040
No (57.1)	10.7 (649)	89.3 (5789)		12.6 (859)	87.4 (6273)	
**Helping a child outside the household regularly, at least once a month**						
Yes (34.6)	9.3 (264)	90.7 (2837)	0.0774	11.1 (486)	88.9 (4077)	0.0075
No (65.4)	10.5 (706)	89.5 (6332)		12.8 (913)	87.2 (6554)	
**Helping someone else outside the household regularly, at least once a month**						
Yes (35.1)	10.6 (323)	89.4 (2960)	0.299	13.6 (617)	86.4 (4165)	0.0008
No (64.9)	9.9 (687)	90.1 (6614)		11.5 (852)	88.5 (6857)	
**Work satisfaction**						
Extremely satisfied (20.2)	3.8 (93)	96.2 (2720)	< 0.0001	5.0 (168)	95.0 (3620)	< 0.0001
Fairly satisfied (56.8)	6.2 (467)	93.8 (7965)		8.7 (836)	91.3 (9300)	
Neither satisfied nor dissatisfied (15.1)	15.3 (357)	84.7 (2069)		19.3 (465)	80.7 (2008)	
Fairly/extremely dissatisfied (7.9)	33.7 (387)	66.3 (811)		36.4 (490)	63.6 (895)	
**Mental strain of work**						
Low (13.8)	4.4 (90)	95.6 (2212)	< 0.0001	6.0 (112)	94.0 (2024)	< 0.0001
Moderate (71.7)	7.9 (737)	92.1 (9602)		9.7 (1160)	90.3 (11,622)	
High (14.5)	24.4 (460)	75.6 (1561)		26.0 (663)	74.0 (1974)	
**Physical strain of work**						
Low (52.8)	8.3 (582)	91.7 (7040)	< 0.0001	10.4 (926)	89.6 (8549)	< 0.0001
Moderate (40.5)	9.2 (524)	90.8 (5573)		11.8 (775)	88.2 (6136)	
High (6.7)	19.1 (187)	80.9 (853)		21.0 (226)	79.0 (874)	
**When I come home, I stop thinking about my work**						
Yes (56.3)	6.9 (492)	93.1 (7440)	< 0.0001	8.1 (787)	91.9 (9621)	< 0.0001
No (43.7)	13.0 (814)	87.0 (6116)		16.9 (1168)	83.1 (6185)	
**I feel that I am neglecting domestic issues because of my work**						
Yes (37.8)	15.5 (761)	84.5 (4583)	< 0.0001	18.5 (1221)	81.5 (5729)	< 0.0001
No (62.2)	6.3 (543)	93.7 (8957)		7.3 (729)	92.7 (10,053)	
**I sometimes ignore my family when I am wholly absorbed in my work**						
Yes (26.4)	14.4 (549)	85.6 (3625)	< 0.0001	18.9 (786)	81.1 (3622)	< 0.0001
No (73.6)	7.8 (751)	92.2 (9883)		9.3 (1160)	90.7 (12,146)	
**I often find it difficult to concentrate on my work because of domestic issues**						
Yes (9.9)	28.7 (419)	71.3 (1106)	< 0.0001	34.5 (556)	65.5 (1118)	< 0.0001
No (90.1)	7.3 (881)	92.7 (12,425)		9.1 (1395)	90.9 (14,651)	
**I have more energy to be with the children when I also go to work**						
Yes (35.1)	6.9 (350)	93.1 (4928)	< 0.0001	9.8 (563)	90.2 (5423)	< 0.0001
No (64.9)	11.1 (936)	88.9 (8425)		12.7 (1362)	87.3 (10,147)	
**I feel inadequacy as a parent**						
Yes (17.8)	20.6 (439)	79.4 (1824)	< 0.0001	20.8 (686)	79.2 (2756)	< 0.0001
No (82.2)	7.7 (850)	92.3 (11,546)		9.4 (1243)	90.6 (12,830)	

Calculated using the inverse probability weights (see Methods)

*MHI-5* Mental Health Inventory

^a^At-risk drinking is assessed as AUDIT-C, men > = 6 points and women > = 5 points

### Separate multivariable logistic regression analyses for men and women

Work-family conflicts were similarly either protective or risk factors in both genders (Table 4). Only one domain, ‘I sometimes ignore my family when I am wholly absorbed in my work’, was associated with psychological distress in women, but not in men. The most distressing domain in both genders was family-to-work conflict, ‘I often find it difficult to concentrate on my work because of domestic issues’, while the second most distressing was ‘I feel inadequacy as a parent’.

**Table 4:** Logistic regression analysis (odds ratios with 95% confidence intervals) of non-work-related and work-related factors with psychological distress (MHI-5 cut-off <=52 points) in the working-age population. Variables were included in the model simultaneously

	Men (*N* = 15,357)		Women (*N* = 18, 271)	
	OR (95% CI)	*P* value	OR (95% CI)	*P* value
Sociodemographic factors				
Age (years)				
20–34	1.28 (0.86–1.92)	0.23	1.39 (0.97–2.00)	0.07
35–49	1.07 (0.75–1.53)	0.72	1.16 (0.84–1.60)	0.38
50–65	1.00		1.00	
Education (years)				
< =12	0.79 (0.56–1.11)	0.18	1.09 (0.80–1.49)	0.58
13–16	0.90 (0.67–1.23)	0.51	0.98 (0.77–1.26)	0.89
17+	1.00		1.00	
Marital status				
Married/cohabiting	1.00		1.00	
Divorced/separated/widowed	1.02 (0.60–1.71)	0.95	1.09 (0.75–1.57)	0.65
Single	1.33 (0.77–2.33)	0.31	1.03 (0.68–1.56)	0.88
Other not work-related factors				
Lives alone	0.71 (0.42–1.22)	0.21	1.26 (0.83–1.91)	0.28
Has children under 18 years of age	**0.69 (0.50–0.96)**	**0.0075**	0.79 (0.58–1.07)	0.13
At-risk drinking^a^	**1.40 (1.10–1.78)**	**0.0057**	1.16 (0.92–1.46)	0.21
Current smoker	**1.46 (1.09–1.94)**	**0.0103**	**1.40 (1.09–1.81)**	**0.0091**
Lonely quite often or all the time	**6.20 (4.17–9.20)**	**< 0.0001**	**6.06 (4.51–8.13)**	**< 0.0001**
Active member in societies, hobby groups, etc.	0.88 (0.67–1.16)	0.37	**0.68 (0.53–0.88)**	**0.0034**
Difficulty in covering household costs	**1.68 (1.31–2.16)**	**0.0001**	**1.70 (1.36–2.11)**	**< 0.0001**
How many persons give you practical help?				
No one	1.00		1.00	
1–2 persons	**0.40 (0.20–0.81)**	**0.01**	0.72 (0.34–1.53)	0.39
3–6 persons	**0.35 (0.17–0.71)**	**0.004**	0.62 (0.28–1.35)	0.23
How many persons give you emotional support?				
No one	1.00		1.00	
1–2 persons	0.83 (0.31–2.22)	0.72	0.41 (0.15–1.14)	0.09
3–6 persons	0.51 (0.19–1.43)	0.20	**0.34 (0.12–0.97)**	**0.04**
Helping parents outside the home	0.78 (0.61–1.01)	0.0569	1.06 (0.86–1.32)	0.57
Helping children outside the home	0.82 (0.62–1.09)	0.17	0.93 (0.74–1.17)	0.55
Helping someone else outside the home	1.05 (0.80–1.38)	0.70	1.20 (0.96–1.50)	0.11
Work-related factors				
Work satisfaction				
Extremely satisfied	1.00		1.00	
Fairly satisfied	**1.70 (1.11–2.60)**	**0.01**	**1.74 (1.24–2.44)**	**0.001**
Neither satisfied nor dissatisfied	**2.96 (1.88–4.66)**	**< 0.0001**	**2.35 (1.58–3.51)**	**< 0.0001**
Fairly/extremely dissatisfied	**6.61 (4.14–11.55)**	**< 0.0001**	**6.08 (4.09–9.04)**	**< 0.0001**
Mental strain of work				
Low	1.00		1.00	
Moderate	1.31 (0.84–2.03)	0.23	0.97 (0.68–1.41)	0.89
High	**2.71 (1.66–4.41)**	**0.0001**	1.34 (0.88–2.04)	0.17
Physical strain of work				
Low	1.00		1.00	
Moderate	0.90 (0.69–1.18)	0.46	0.84 (0.66–1.05)	0.13
High	1.19 (0.78–1.80)	0.43	0.86 (0.57–1.28)	0.45
When I come home, I stop thinking about my work^b^	**0.64 (0.50–0.83)**	**0.0008**	**0.58 (0.46–0-73)**	**< 0.0001**
I feel I am neglecting domestic issues because of my work^b^	**1.53 (1.14–2.04)**	**0.004**	**1.60 (1.25–2.04)**	**0.0002**
I sometimes ignore my family when I am wholly absorbed in my work^b^	0.94 (0.69–1.27)	0.67	**1.30 (1.00–1.70)**	**0.0497**
I often find it difficult to concentrate on my work because of domestic issues^b^	**3.24 (2.42–4.32)**	**0.02**	**2.70 (2.08–3.49)**	**< 0.0001**
I have more energy to be with the children when I also go to work^b^	**0.72 (0.54–0.95)**	**< 0.0001**	**0.77 (0.59–0.99)**	**0.047**
I feel inadequacy as a parent^b^	**2.21 (1.64–2.98)**	**0.03**	**2.04 (1.57–2.64)**	**< 0.0001**

Bold ratios: statistically significant results

Variables were included in the model simultaneously

*OR* Odds ratio, *95% CI* 95% confidence interval

^a^At-risk drinking is assessed as AUDIT-C, men > = 6 points and women > = 5 points

^b^N indicates the number of yes answers

Being fairly or extremely dissatisfied with work had the strongest association with on psychological distress measure in both genders. The high mental strain of one’s work had a statistically significant association with psychological distress only in men, whereas the physical strain of one’s work was not associated with distress in either gender.

Of the other variables considered, loneliness was strongly associated with psychological distress among both genders. Smoking and difficulties in covering household costs were also similarly associated with psychological distress in both genders. With respect to other not work-related factors, having minor children and actively participating in hobby groups and societies were associated with lower odds, while feeling inadequacy as a parent was associated with higher odds for psychological distress.

Having someone to give practical help (among men) or emotional support (among women) when needed were both associated with lower odds of psychological distress, especially when several supporters were available. Helping others outside the home was not associated with psychological distress.

### Combined logistic regression analysis to test for gender interactions

In the logistic regression models conducted separately for men and women, gender difference in the strength of the associations with psychological distress appeared possible in the following variables: having children under 18 years old, at-risk drinking, active participation, receiving practical help from others, receiving emotional support from others, mental strain of work and ignoring family when wholly absorbed in one’s work. We included these variables in the logistic regression model pooled together across gender to test if they exhibited statistically significant gender interaction.

We found significant interaction by gender in two variables. The interaction term ‘gender and mental strain’ proved significant (F value 3.86, *p* = 0.0212), indicating that mental strain was associated with psychological distress in men (see Table 4). The interaction term ‘gender and ignoring family due to being absorbed in one’s work’ also proved significant (F value 4.16, *p* = 0.0414), indicating that ignoring family due to being absorbed in one’s work was associated with psychological distress in women.

## Discussion

In this cross-sectional, nationally representative study sample of the working population, we found that several factors related to work and balancing work and family life are associated with psychological distress. Furthermore, these associations were mostly similar among women and men. Earlier studies have yielded mixed evidence regarding gender differences based on work-family conflicts [24].

Psychological distress is quite common problem. In the current study, 11% of women and 8.8% of men in the working population had psychological distress. In the most recent national FinSote Survey from years 2017–2018, where participants were over 19 years with no upper age limit, the prevalence of psychological distress among women was 11.9% and among men 11.2% [83], suggesting that people who are employed full-time may experience slightly less psychological distress than the rest of the population. In large surveys made in the United States, 15.1% reported moderate psychological distress and 3.1% severe distress over the 2001–2012 period [84]. Because of the different rating scales and cut-off scores used in previous studies, the reported prevalence figures of psychological distress are not directly comparable between countries. With the cut-off score used in the current study, some underlying mood or anxiety disorder is very probable [1].

Family-to-work conflict has previously been found to be less common than work-to-family conflict [85], but in our study family-to-work conflict was more strongly associated with psychological distress than work-to-family conflict. The only gender difference was found in sometimes ignoring family when wholly absorbed in one’s work, which was associated with psychological distress only in women. This suggests that an engaging job may cause psychological distress via work-to-family conflict among women [86]. Difficulty in concentrating on work because of domestic issues showed the strongest association with psychological distress, but it could also imply that the participants were experiencing distressing family-related challenges at the time.

We also found evidence of work-to-family enrichment: those who responded that they have more energy to be with their children when they also go to work had less psychological distress [15]. Also, participants who reported that they stop thinking about their work when they come home had less distress, suggesting that a successful combination of work and family life protects a person from psychological distress. Inadequacy as a parent was associated with psychological distress independently of gender. Prior studies have reported that about half of employed parents feel they do not spend enough time with their children, and such a time deficit is associated with psychological distress [87].

Interestingly, mental strain of one’s work was a risk for psychological distress in men but not in women. The link between the mental demands of one’s work and psychological distress or mental disorder has been observed both in cross-sectional and in longitudinal studies [87–90]. Emotional exhaustion is more common in emotionally demanding jobs, such as in police work or among physicians and other professionals working in healthcare, and effective preventive interventions are available [91]. Most previous studies have not found any gender difference in how the psychological or emotional demands of one’s work affect mental health, but one previous study found that they may have a mediating effect between low income and psychological distress in men [92].

Consistent with prior studies [27, 93], our findings showed that job dissatisfaction was strongly associated with psychological distress. We did not find any gender difference in terms of job dissatisfaction, which is consistent with earlier studies [29, 94]. In a meta-analysis on the health effects of job dissatisfaction, the strongest correlation between job dissatisfaction and mental health problems was with burnout [27]. Burnout is a chronic stress syndrome characterised by exhaustion, cynicism and a lack of professional efficacy [95], and it may be an important mediator in the observed association between job dissatisfaction and psychological distress.

Loneliness was, similar to job dissatisfaction, the most significant factor increasing the odds of psychological distress, and at the same magnitude, in both genders. Women reported feelings of loneliness more often than men, as has been found earlier [41], but the association with psychological distress was equal in both genders. Previous studies have shown that loneliness is a significant risk factor for depression [96] and other common mental disorders [43] as well as for suicidal ideation and suicide attempts [97]. Furthermore, loneliness is associated with an increased risk of many health problems [97], and it has been increasingly seen as an important public health problem [98]. Our finding supports this view and encourages experts to implement specific interventions to reduce loneliness [99].

Various aspects related to social networks and social support were associated with having less psychological distress. Having minor children, being active in hobby groups, and receiving social support when needed were all associated with less psychological distress. Previous studies have found that social participation in activities is especially beneficial for women [33, 38], whereas men and women benefit differently from emotional support [33–35]. However, while the analyses conducted separately among men and women suggested that there might be gender differences in these aspects of social networks and support, we did not observe any significant interaction in the analysis. It is also noteworthy that helping others outside the home was not associated with psychological distress.

Previous studies [66, 68, 70] have likewise found that financial difficulties constitute a notable risk factor for psychological distress. Consistent with a large body of previous research, smoking [59, 100, 101] and at-risk drinking [102, 103] were associated with more psychological distress; however, their effect was less prominent than that of social and work-related factors in our study.

After considering a wide range of work-related, family-related and social factors, well-known risk factors like marital status and living alone did not have an association with psychological distress. This is consistent with a previous study that found that loneliness is a mediator between living alone and distress [104].

### Strengths and limitations

The major strength of the present study is the large study sample representative of the adult working-age population in Finland. It was possible for participants to respond in several languages spoken by sizeable minorities within Finland, which further improved the representativeness. The Finnish version of MHI-5 has been shown to have construct validity [105].

The major limitation is that our study is cross-sectional. Therefore, we could not assess the direction or causality of the associations. Furthermore, data were obtained using self-report questionnaire and therefore we did not get detailed information e.g. about mental disorders. Self-reporting bias, such as social desirability bias and recall bias, could affect the results [106]. We did not have information about job demands, job control or other features related to work. Low response rate is a common phenomenon in survey studies today, and so it was in our study as well, especially for the youngest age group. However, we used inverse probability weighting to account for missing data, which has been shown to remove a relatively large proportion of the bias related to the low response rate in the current study sample [76]. In addition, we did not have information on all factors potentially related to gender differences in mental health. For example, intimate partner violence is strongly associated with psychological distress, and women experience it more often than men [107].

The results may not be generalizable to countries where there is more gender inequality. According to the European Institute for Gender Equality, Finland ranked the fourth in the European Union on the Gender Equality Index [108]. The gender gap in full-time equivalent employment rate and duration of working life is much smaller in Finland than in European countries on average, although there is still a gender gap in mean monthly earnings. There is a gender gap in caring for children, grandchildren or older people and in housework in Finland, but it is smaller than the European average. Women in Finland have higher level of education than men, and in our current parliament there are almost as many women as men [108]. In the Global Gender Gap report, Finland ranked the third [109]. Therefore, it is probable that in another country where gender inequality is more widespread or the culture is less individualistic, these results would be different [110].

## Conclusions

Satisfying work, family life and being able to successfully combine both are important sources of psychological well-being in the working population, both among men and women. Of all studied variables, loneliness and being dissatisfied with work were most strongly associated with psychological distress. The detrimental effect of loneliness on mental health has become apparent during the COVID-19 pandemic [111] and should receive more attention in mental health policies and promotion. The strong association between dissatisfaction with work and psychological distress is likely bidirectional, and underscores the need to improve workplace mental health literacy and the availability of mental health interventions [112].

## Supplementary Information

**Additional file 1.**

## Data Availability

The data that support the findings of this study are not publicly available. The data, from which all personal data have been eliminated, may be disclosed for research purposes by FinData in return for a research proposal and an approved user authorisation application.

## Abbreviations

ATH: Finnish Regional Health and Well-being Study
MHI-5: Mental Health Inventory-5
OR: Odds ratio
AUDIT-C: The Alcohol Use Disorders Identification Test
CI: Confidence interval
