# Supplementary figures and images for: Factors contributing to psychological distress in the working population, with a special reference to gender difference
Source: BMC Public Health. 2021 Mar 29;21:611. doi: 10.1186/s12889-021-10560-y (PMC8006634; doi:10.1186/s12889-021-10560-y)

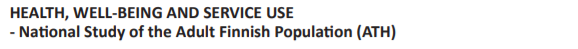


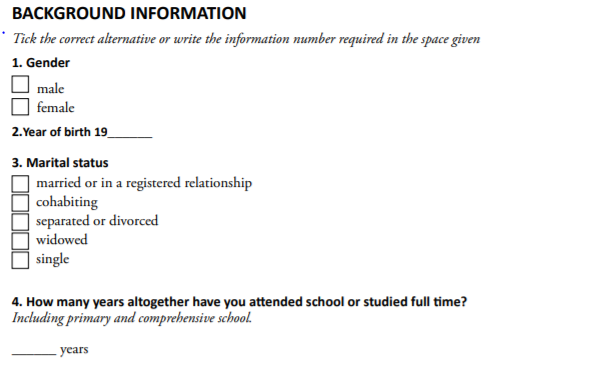


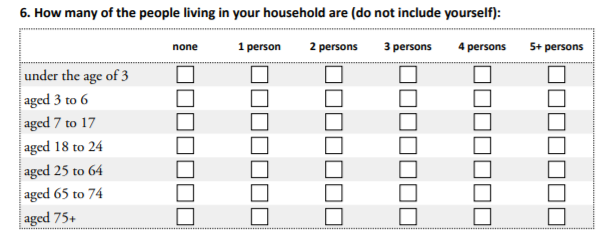


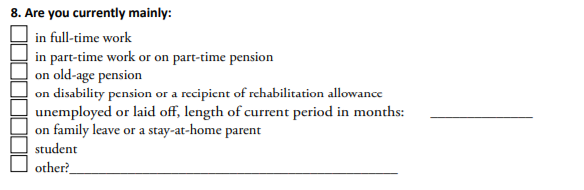


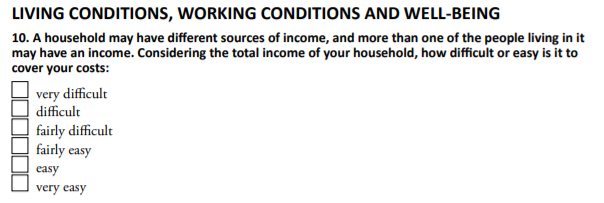


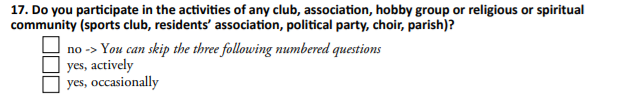


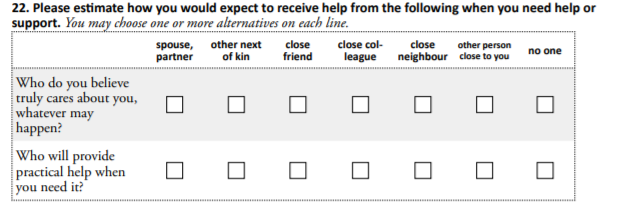


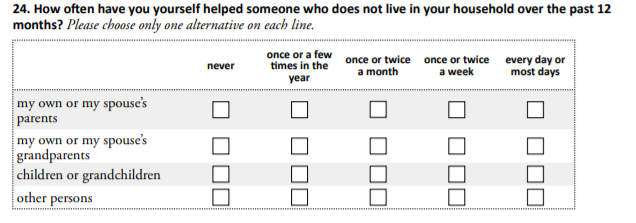


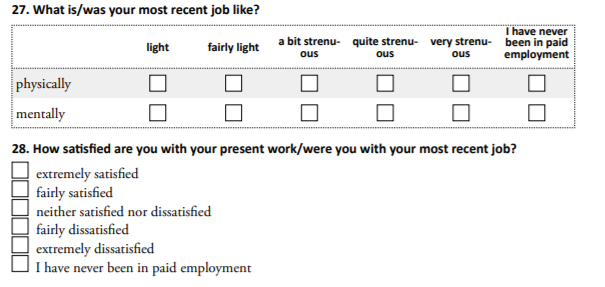


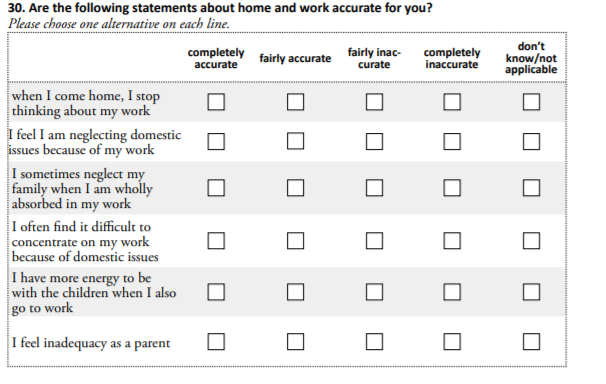


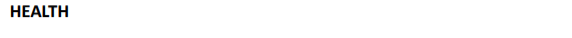


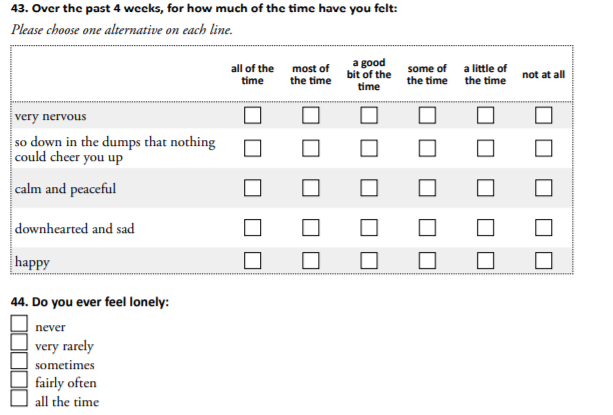


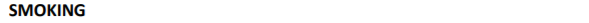


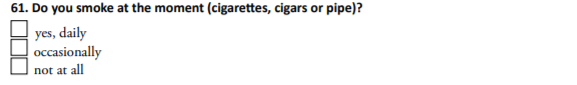


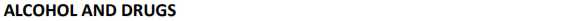


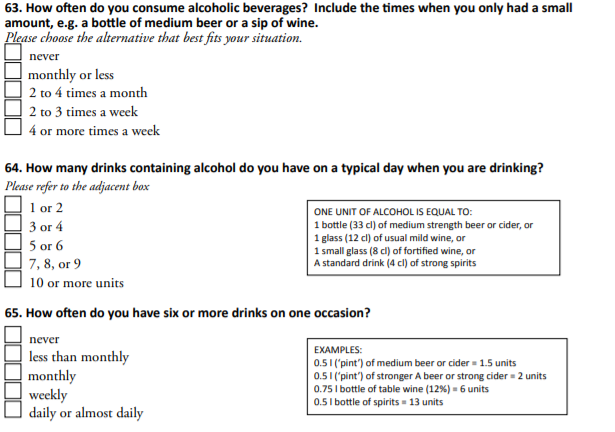

Supplement: Supplementary file 1 — Additional file 1. [file 12889_2021_10560_MOESM1_ESM.docx]
